# Anti-Tumor Activity of AZD4547 Against NTRK1 Fusion Positive Cancer Cells Through Inhibition of NTRKs

**DOI:** 10.3389/fonc.2021.757598

**Published:** 2021-11-01

**Authors:** Hanna Cho, Namkyoung Kim, Takashi Murakami, Taebo Sim

**Affiliations:** ^1^ KU-KIST Graduate School of Converging Science and Technology, Korea University, Seoul, South Korea; ^2^ Severance Biomedical Science Institute, Graduate School of Medicinal Science, Brain Korea 21 Project, Yonsei University College of Medicine, Seoul, South Korea; ^3^ Department of Microbiology, Saitama Medical University, Saitama, Japan

**Keywords:** drug repositioning, AZD4547, NTRK fusion, NTRK inhibitor, colorectal cancer, Ba/F3 TRK

## Abstract

Inhibitors of tropomyosin-related kinases (TRKs) display remarkable outcomes in the regression of cancers harboring the Neurotrophin Receptors Tyrosine Kinase (NTRK) fusion gene. As a result, TRKs have become attractive targets in anti-cancer drug discovery programs. Here, we demonstrate that AZD4547, a highly potent and selective inhibitor of fibroblast growth factor receptor (FGFR), displays anti-tumor activity against KM12(Luc) harboring the TPM3-NTRK1 fusion gene associated with its direct inhibition of TRKs. The results of profiling, using a 64-member in-house cancer cell panel, show that AZD4547 displays anti-proliferation activity against KM12(Luc) with a GI_50_ of 100 nM. *In vitro* biochemical assays reveal that AZD4547 has IC_50_ values of 18.7, 22.6 and 2.9 nM against TRKA, B and C, respectively. In a cellular context, AZD4547 blocks auto-phosphorylation of TRKs and phosphorylation of its downstream molecules including PLC-gamma and AKT in a dose dependent manner. Also, AZD4547 at 0.1 μM concentration downregulates expression of MAPK target genes (DUSP6, CCND1 and ETV1) as well as the E2F pathway. Furthermore, AZD4547 induces G0/G1 arrest and apoptosis, and suppresses anchorage independent growth of KM12(Luc). Oral administration of 40 mpk AZD4547 dramatically delays tumor growth in a KM12(Luc) implemented xenograft model, without promoting body weight changes. The capability of AZD4547 to inhibit TRKA, TRKB and clinically relevant mutants (TRKA G595R, G667S, G667C and G667A) was also evaluated using Ba/F3 cells harboring the ETV6-NTRKs fusion gene. The combined observations demonstrate the potential application of AZD4547 for treatment of NTRK fusion driven cancers.

## Introduction

Receptor tyrosine kinases are among the most frequently dysregulated proteins involved in cancers (1). As a result, these kinases have become key drug targets in studies conducted during the past two decades. Thus far (2021), 62 kinase inhibitors have been approved, among them 52 kinase inhibitors are Tyrosine kinase inhibitors (TKI) (2). The intense effort in this area has also led to the development of many TKIs that were unsuccessful in clinical trials mainly due to insufficient efficacy for humans. However, second opportunities exist for the failed TKIs as a consequence of drug repositioning, which focusses on discovering new applications of existing therapeutic agents (3, 4). Because substances can interact with more than one target protein (5), they have the potential of having more than one pharmacological function. Furthermore, owing to its relatively low cost and risk, and time-saving nature, drug repositioning is an effective strategy in drug development. So far, over 14 existing drugs have been successfully repositioned and several promising candidates are in different stages of clinical trials exploring their new uses (6).

Neurotrophin receptors tyrosine kinase (NTRK) genes encode Tropomyosin-related kinases, which are receptors for different neurotrophins [TRKA is for NGF, TRKB is for BDNF and NT4, and TRKC is for Neurotrophin3 (NT3)] (7, 8). Chromosomal rearrangement of NTRKs with upstream partner genes including TPM3, ETV6 and LMNA leads to oncogenic TRK activation (8). The NTRK gene fusions are found in various tumor patients across histologies that include colorectal cancer, NSCLC, thyroid carcinomas, and glioblastoma (9, 10). Although NTRK fusion genes occur in less than 1% of all solid tumors, TRK inhibitors display remarkable therapeutic responses (11). Therefore, several TRKA/B/C inhibitors have been developed and subjected to clinical trials (12–24). Among this group, LOXO101 (larotrectinib, VITRAKVI^®^), a potent and selective TRKs inhibitor, and entrectinib (Rozyltrek^®^) have been recently approved in the U.S.A and Japan, respectively, for the treatment of patients who have a NTRK gene fusion, regardless of the cancer type (25–27). The unusual FDA approval for LOXO101 demonstrates the importance of TRKA/B/C in terms of targeted anticancer therapy.

AZD4547, first developed as a potent fibroblast growth factor receptor (FGFR) inhibitor, exhibits remarkable *in vitro* potencies against FGFRs 1–3 (IC_50_ = 0.2 nM for FGFR1, 2.5 nM for FGFR2, and 1.8 nM for FGFR3). In addition, it is highly selective against FGFR1-3 in that AZD4547 inhibits only two other kinases (Insulin-Like Growth Factor 1 Receptor (IGFR1) and Kinase Insert Domain Receptor (KDR) at sub-μM IC_50_s (28). The recent phase II clinical trials of AZD4547 for the patients with FGFR genetic mutations are completed (NCT02465060) with failure to meet the primary end point. Currently, phase 1b study of multi-drugs including AZD4547 (NCT02546661) is undergoing for the Muscle Invasive Bladder Cancer (29). Although a number of clinical trials have shown that AZD4547 has acceptable safety, tolerability and a moderate efficacy (30–33), they have not yet led to FDA approval. These failures have stimulated efforts to seek new applications of AZD4547. Unlike the diverse number of studies that have focused on the anti-tumor properties of AZD4547 against FGFRs-deregulated tumor cell lines (34), the investigation described below was aimed at exploring new targets by testing the anti-tumor effects of this substance that are not related to FGFR mutations. The results of this study, in which we assessed the anti-proliferative activity of AZD4547 on 64 cancer cell lines having different mutational status, demonstrated that AZD4547 potently suppresses proliferation of KM12(Luc), which has the TPM3-NTRK1 fusion gene that drives colon cancer (35), and Ba/F3 harboring TRKA/B. Moreover, AZD4547 has a comparable *in vivo* efficacy in the KM12(Luc) tumor xenograft model. Finally, the results of this study show for the first time that AZD4547 directly inhibits TRKA/B/C activity as well as FGFRs.

## Materials and Methods

### Reagents

Inhibitors, including AZD4547 (MedChemExpress, HY-13330) were purchased from the indicated companies. For use in the apoptosis assay, Alexafluor488 conjugated annexin V (A13201) and propidium iodide (P3560) were purchased from Thermo Fisher Scientific. For use in cell cycle analysis, propidium iodide in a RNase containing solution (#4087) was obtained from Cell Signaling Technology. Primary antibodies including phospho-TRKA/B, phospho-AKT (Ser473, #4058), phospho-ERK1/2 (Thr202/Tyr204, #4370), ERK1/2 (#4695), phosphor-PLC-gamma (Tyr783, #2821) and PARP-1 (#9542) were purchased from Cell Signaling Technology. β-actin (SC-47778), DUSP6 (SC-377070) and phospho-MEK1/2 (Ser218/222, SC-81503) were purchased from Santa Cruz Biotechnology. AKT (A18120) and MEK (A4868) were obtained from Abclonal. Secondary antibodies were purchased from genDEPOT. In the colony formation assay, colonies are stained with iodonitrotetrazolium chloride (Sigma Aldrich, #I8377).

### Cell Culture

KM12(Luc) was purchased from the JCRB cell bank. KM12 (Luc) was used within 6 months after purchase and the DNA profiles were confirmed using a short tandem repeat (STR) analysis by the Korean Cell Line Bank. KM12(Luc) cells were maintained in DMEM supplemented with 10% FBS and antibiotics. Parental Ba/F3 cells were purchased from DSMZ and were grown in RPMI1640 supplemented with 10% FBS and antibiotics in the presence of IL3, Transformed Ba/F3 cell lines were grown in the same media without IL3. Cells used in this study were negative for mycoplasma contamination. AN3-CA, HEC1A, U138, 8505C, MKN28, MKN45, A172, A375, Capan-1 HeLa, HEP3B, MCF-7 and MIA-paca-2 cells were maintained in DMEM (Welgene, Korea) supplemented with 10% FBS and antibiotics (Welgene, Korea). Otherwise, all cells were maintained in RPMI (Welgene, Korea) supplemented with 10% FBS and antibiotics (Welgene, Korea).

### Cell Viability Test

To obtain the GI_50_ value of a substance, cell viability was measured using MTT (G4000, Promega, USA) for KM12(Luc) cell and Cell titer glo (G7572, Promega, USA) for other cell lines. For adherent cells, cells were seeded at 5,000 cells/well in a 96-well plate and treated with a substance one day after cell seeding. For suspension cells, 10,000 cells/well were seeded and the substance was treated after 4 h stabilization of cells. The substance was treated at 10 points of 1:3 serial dilution (0–100 µmol/L) for 72 h. For MTT assay, 15 µL dye solution was added to each well and incubated 4 h at 37°C. 100 µL of solubilization solution was added and followed 1 hr incubation. Absorbance at 570 nm of each well was recorded using EnVision microplate reader (Perkin Elmer). For Cell titer glo assay, Cell titer glo reagent was treated in each well and measured the luminescence by EnVision microplate reader (Perkin Elmer). Viable cells in each well were normalized by using 0.5% DMSO-treated wells (100%). The inhibitor dose−response curve was fitted and GI_50_ values were calculated using Prism 7.0 software (GraphPad). All assays were performed in duplicate and the standard deviation (SD) was determined from two independent experiments.

### Establishment of Ba/F3 Stable Cell Lines (ETV6-NTRKs)

To establish Ba/F3 cells expressing ETV6 fused TRKA or TRKB kinase domains, plasmids containing TEL-fused human NTRK1/2 kinase domain were inserted by retrovirus infection. For mutagenesis PCR, QuikChange II Site-Directed Mutagenesis Kit (#200523, agilent) was used. The primers used for establishing point mutation are as follows: TRKA G595R (g1783a) 5-agtatatgcggcacagggacctcaaccgc-3, 5-gcggttgaggtccctgtgccgcatatact-3; TRKA G667A (g2000c) 5-gactggtggtcaagattgctgattttggcatgagc-3, 5-gctcatgccaaaatcagcaatcttgaccaccagtc-3; TRKA G667S (g1999a) 5-ggactggtggtcaagattagtgattttggcatgagc-3, 5-gctcatgccaaaatcactaatcttgaccaccagtcc-3; TRKA G667C (g1999t) 5-ggactggtggtcaagatttgtgattttggcatgagc-3, 5-gctcatgccaaaatcacaaatcttgaccaccagtcc-3.

### Immunoblot Analysis

Cells treated under the indicated condition were briefly washed with ice-cold DPBS. Thereafter, cell lysates were prepared by using a buffer solution containing 50 mM Tris-HCl pH7.5, 1% NP40, 1 mM EDTA, 150 mM NaCl, 5 mM Na_3_VO_4_, 2.5 mM NaF and a protease inhibitor cocktail (Roche, #11873580001). The same amount of lysate was separated by using SDS-PAGE gel and transferred to the PVDF membrane. All primary antibodies were uniformly prediluted in TBS containing 0.05% Tween 20 at 1:1000 (v/v) whereas actin and all secondary antibodies were prediluted in TBS containing 0.05% Tween 20 at 1:5000.

### RT-PCR

Total RNAs of each cell were extracted by using TRIzol reagent (Invitrogen) and 2 µg of total RNA was used to create cDNA using M-MLV reverse transcriptase (Promega). The same amount of cDNA was amplified by using PCR Cell safe premix (Cat#) for 32 cycle. Amplified PCR products were confirmed by using electrophoresis in 3% agarose gels and visualized by Eco-star dye (Biofect #ES301-1000). The primer sequences used in this study are as follows: CCDN1(156bp) (36) 5-cctctgtgccacagatg-3 (forward), 5-gggtcacacttgatcactc-3 (reverse); E2F1 (37) 5-gccactgactctgccaccatag-3 (forward), 5-ctgcccatccgggacaac-3 (reverse); DUSP6 (38) 5-gagtctgaccttgaccgagaccccaa-3 (forward), 5-ttcctccaacacgtccaagttggtggagtc-3 (reverse); ETV1 (39) 5-taccccatggaccacagatt-3 (forward), 5-cactgggtcgtggtactcct-3 (reverse); FOSL1 (40) 5-tgaccacaccctccctaactc-3 (forward), 5-ctgctgctactcttgcgatga-3 (reverse); gapdh (41) 5-ggtggtctcctctgacttcaaca-3 (forward), 5-gttgctgtagccaaattcgttgt-3 (reverse); FGFR1 (41) 5- cgcccctgtacctggagatcatca-3(forward), 5- ttggtaccactcttcatctt-3; FGFR2 (41) 5- gcctggaagagaaaaggagattac-3 (forward), 5- actgtacaccttgcagtgga -3(reverse); ETV6 5-atgatccgccgcctctcc-3 (forward); NTRK1 5- tggtggctgtcaaggcactg -3 (forward), 5- gatctcccagagcaccacgc-3 (reverse); NTRK2 5- cacgcaaggacttccaccgt -3 (forward), 5- ccaaatcgcggtgcacgaag-3 (reverse).

### Apoptosis and Cell Cycle Analysis

For apoptosis assays, cells treated with under the indicated condition were briefly washed with ice-cold DPBS. Thereafter, the trypsinized cells were stained with Alexafluor488 conjugated annexin V and propidium iodide according to the manufacturer’s instructions. Apoptotic cells were monitored by using Accuri C6 (BD Biosciences, USA). For cell-cycle analysis, cells treated under the indicated condition were washed with ice-cold DPBS. Thereafter, the trypsinized cells were fixed in 70% ethanol at −20°C overnight. The cells were centrifuged at 500 g for 10 min followed by twice washing with ice-cold DPBS, and then stained with propidium iodide/RNase solution for 30 min. The DNA content of each cell was determined by using Accuri C6 (BD Biosciences, USA).

### Anchorage Independent Assay

On the 1.6% bottom agar, 10,000 cells in the complete media containing 0.8% agar were plated in 6-well plates. Indicated substances in complete media were added in the top agar and media and substances were refreshed twice a week. After 2 weeks incubation, colonies from each well were stained with iodonitrotetrazolium chloride (Sigma-Aldrich) for 24 h. The entire area of each well was photographed and the colonies were counted by using ImageJ software.

### Xenograft Model

All animal procedures were approved by the KIST Laboratory Animal Facilities and Use Committee and carried out in AAALAC-accredited facilities. KM12(Luc) cells (2.5 × 10^6^ cells/0.1 mL) were subcutaneously implanted into the right flank of 7-week-old female balb/c nude mice (Orient Bio Inc.) When the tumor volume reached around 100 mm^3^, the tumor-bearing mice were randomly separated into 4 cohorts (n = 4 per cohort). The vehicle substances are follows: 5% NMP, 15% solutol, 30% PEG400 and 50% 0.05M citric acid. Mice were orally gavaged once daily for Vehicle, AZD4547 or twice per day for LOXO195 for 14 d. Tumor volume and body weight were measured once every 2 d. The formula used to calculate tumor volumes is tumor volume = 1/2 (length × width^2^)

### Statistical Analysis

All data were analyzed with Graphpad prism version 7.0.0 for window, Graphpad software, San Diego, California USA. Statistical analysis used for each dataset is stated in the figure legend. Standard error of mean (S.E.M) or standard deviation (s.d) are indicated in each figure legends.

## Results

### Anti-Proliferation Activity of AZD4547 on KM12(luc) Through Inhibition of NTRK

**Figure 1:**
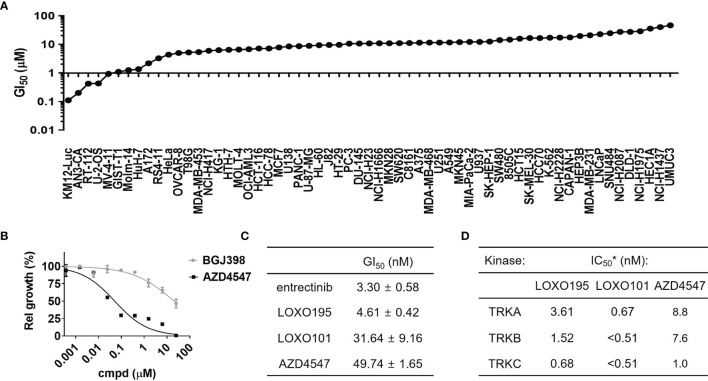
AZD4547 is pan-TRK inhibitor. **(A)** Results of cancer cell screening of AZD4547 against 64 cancer cell lines. GI_50_ of AZD4547 against 64 cancer cell lines are measured after 72 h incubation of serial diluted AZD4547. **(B)** Anti-proliferation activity of AZD4547 and BGJ398 on KM12(Luc). Cell viability of KM12(Luc) after 72 h exposure to AZD4547 or BGJ398 were determined by using a cell titer glo assay. **(C)** GI_50_ values of TRK inhibitors and AZD4547 against KM12(Luc). GI_50_ values were determined by using an MTT assay after 72 h exposure to the indicated substances. All experiments are duplicate mode and each GI_50_ is an average of two independent assays. **(D)** IC_50_ of AZD4547 against TRK isotypes. *Each of IC_50_ was obtained by using a radiometric biochemical kinase assay.

To identify AZD4547 sensitive cell lines other than FGFR-mutant driven cancer cells, GI_50_ values were determined for AZD4547 against 64 different cancer cell lines, including AN3-CA used as a positive control (**Figure 1A** and **Table S1**). Out of the 64 cell lines five including KM12(Luc), AN3-CA, RT-112, U-2-OS and MV4-11 have GI_50_ values for AZD4547 that are <1 µM. Among this group, RT-112 harboring high expression of FGFR3 is a previously reported cell line in which AZD4547 is a sensitive inhibitor (100-200 nM of GI_50_) (42). Also, inhibitory activity of AZD4547 against DDR1 or FLT3 allows suppression of proliferation of U-2-OS and MV4-11 (43). Therefore, our attention focused on the inhibitory activity of AZD4547 against KM12(Luc) cell lines which have the TPM3-NTRK1 fusion gene. To exclude the possibility that the anti-proliferation activity of AZD4547 against KM12(Luc) is a common characteristic of FGFR inhibitors, we determined if BGJ398, a representative FGFR inhibitor, is also capable of suppressing proliferation of KM12(Luc) cells (**Figure 1B**). The results show that BGJ398 has no inhibitory activity against proliferation of KM12(Luc) cell lines indicating that the inhibitory effect on KM12(Luc) is a unique feature of AZD4547. Next, the anti-proliferation activity of AZD4547 on KM12(Luc) was compared with those of the representative TRK inhibitors entrectinib, LOXO101 and LOXO195 (**Figure 1C**). The result shows that AZD4547 has a comparable potency (GI_50_ = 49.74 nM) to that of LOXO101 (GI_50_ = 31.64 nM). To determine whether AZD4547 directly inhibits TRK kinase activity, we performed an *in vitro* kinase assay against TRKs (**Figure 1D**). AZD4547 shows great *in vitro* potencies with IC_50_ = 8.8 nM against TRKA, 7.6 nM against TRKB and 1.0 nM against TRKC. Considering the respective IC_50_s of AZD4547 against FGFR1, 2 and 3 are 0.2 nM, 2.5 nM and 1.8 nM, the observed inhibitory activity of AZD4547 against TRKC is noteworthy. Compared to the IC_50_s of LOXO101 and LOXO195, AZD4547 displays comparable activity on TRKC to that of LOXO195 (IC_50_ = 0.68 nM against TRKC) although both LOXO101 and LOXO195 exhibit higher activities on TRKA and TRKB than AZD4547.To show that AZD4547 inhibits phosphorylation of TRKs at the cellular level, western blot analysis was conducted (**Supplementary Figure 1A**). the results demonstrate that 1 µM AZD4547 completely suppresses phosphorylation of TRKA/B while BGJ398 and PD173074, other representative FGFR inhibitors, do not inhibit phosphorylation of TRKA/B. This finding indicates that inhibition of TRK by AZD4547 is not related to inhibition of FGFRs. Based on these results, we conclude that AZD4547 inhibits a pan-TRK as well as FGFRs.

### Activity of AZD4547 Against TRKs Mutants

**Figure 2:**
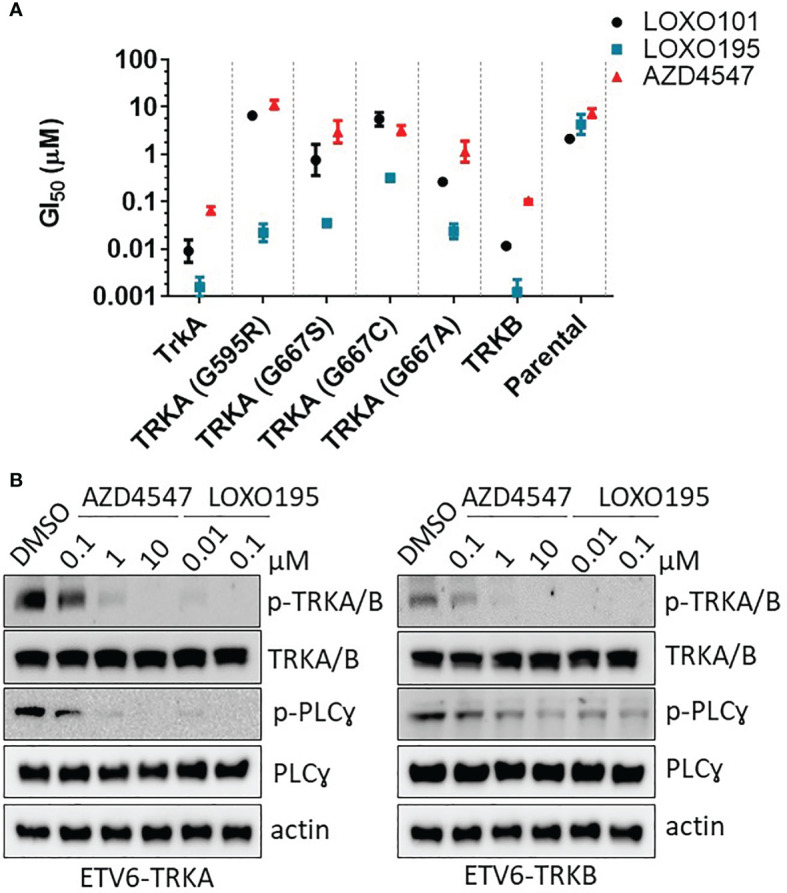
Effects of AZD4547 on TRKA kinase mutants and TRKB. **(A)** Summary of GI_50_s of AZD4547 against Ba/F3: TRKA mutants or TRKB. Cell viability after 72 h exposure to the indicated inhibitors, determined by using a cell titer glo assay in duplicate mode. Data was plotted as mean GI_50_ ± SD of two independent experiments. **(B)** AZD4547 inhibits phosphorylation of TRKA/B and PLC-gamma in Ba/F3 harboring TRKA and TRKB, respectively. The phosphorylation level of TRKA/B and PLC-gamma were monitored after 2 h treatment with AZD44547 or LOXO195. Data are representative of three independent experiments.

To investigate the inhibitory activity of AZD4547 against clinically relevant TRK mutants which have resistance to the known TRK inhibitors (8, 44), we established Ba/F3 cell lines harboring the ETV6-NTRKs fusion genes (NTRK1, NTRK1G595R, NTRK1G667A, NTRK1G667C, NTRK1G667S and NTRK2) (**Supplementary Figure 2**). The mutation status of each established cell line was confirmed by sequencing of its cDNA (**Supplementary Figure 2**). The anti-proliferation activity of AZD4547 was then measured in each cell line (**Figure 2A** and **Table S2**). As expected, AZD4547 significantly inhibits the proliferation of Ba/F3: TRKA (GI_50_ = 66 nM) and Ba/F3: TRKB (GI_50_ = 100 nM). However, AZD4547 has no inhibitory activity against Ba/F3 harboring clinically relevant TRKs mutants including TRKA G595R, TRKA G667S, TRKA G667C and TRKA G667A. We next evaluated if AZD4547 is capable of suppressing the phosphorylation of TRKA/B in Ba/F3 cell lines (**Figure 2B**). Treatment with AZD4547 for 2 h leads to potent dose-dependent inhibition of phosphorylation of TRKA/B and PLC-gamma. Also, phosphorylation of TRKA/B is almost completely abolished using 1 µM of AZD4547. These results indicate that the inhibitory activity of AZD4547 against TRKA/B is well translated into a cellular context.

### AZD4547 Impedes TRKs Signaling Pathway in KM12(Luc)

**Figure 3:**
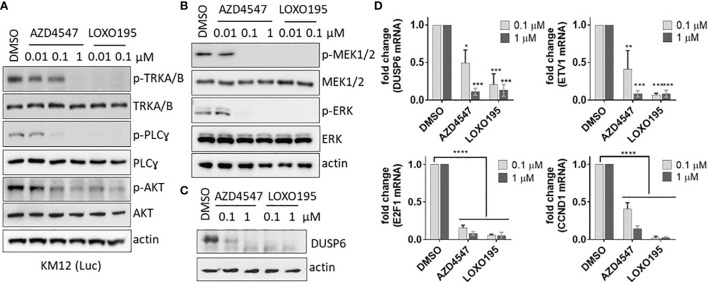
AZD4547 suppresses phosphorylation of TRKA/B and its downstream substances. **(A)** AZD4547 inhibits phosphorylation of TRKA/B, PLC-gamma and AKT in KM12(Luc). The phosphorylation level of TRKA/B, PLC-gamma and AKT were monitored after 2 h treatment with AZD44547 or LOXO195. Data are representative of two independent experiments. **(B)** AZD4547 decreases MAPK signaling in KM12(Luc). AZD4547 or LOXO195 treated for 2 h against KM12(Luc). Level of phospho- MEK1/2 and ERK1/2 were evaluated by western blot. Data are representative of two independent experiments. **(C)** AZD4547 treatment reduces the DUSP expression level in KM12(Luc). After 24 h treatment with the indicated substances, the cell lysates were subjected to western blot with DUSP6 antibody. Data are representative of two independent experiments. **(D)** AZD4547 blocks the MAPK and E2F pathways. After treatment of AZD4547 or LOXO195 for 24 h, total RNA was extracted, and cDNA was synthesized. Each cDNA was amplified by using the indicated primer for comparison of mRNA levels. (average ± SEM, n=3, twoway Anova, * < 0.05, ** < 0.005, *** < 0.001, ****p < 0.0001).

Next, the capability of AZD4547 to block the TRK pathway in KM12(Luc) (TPM3-NTRK1) was evaluated. For this purpose, we analyzed the phosphorylation level of TRKA/B and its downstream molecules by using western blotting (**Figure 3A**). Treatment of AZD4547 for 2 h leads to inhibition of TRKA/B phosphorylation in a dose-dependent manner and phosphorylation of TRKA/B is completely abolished at 1 µM of AZD4547. Because the PLC-gamma and Akt pathways are known to be regulated by the TRK pathway (7), we also monitored the phosphorylation levels of the PLC-gamma and Akt. In the presence of AZD4547, phosphorylation of PLC-gamma and Akt decreases in an AZD4547 dose-dependent manner. It is noteworthy that inhibition of phosphorylation by AZD4547 is comparable to that by LOXO195, which a promising next-generation TRK Inhibitor. The effect of AZD4547 on MAPK signaling a downstream pathway of TRK was also assessed. The results show that phosphorylation of MEK1/2 and ERK were inhibited completely by 0.1 μM of AZD4547 (**Figure 3B**). Moreover, protein expression of DUSP6, one of the target genes of MAPK, is downregulated by AZD4547 treatment for 24 h (**Figure 3C**). Moreover, the consistent result of DUSP6 expression was observed in RT-PCR assay (**Figure 3D**). AZD4547 treatment for 24 h results in a decrease in mRNA levels of MAPK target genes including ETV1 and DUSP6. Because expression of E2F and CCND1 are also down-regulated by TRK inhibitors (18), we monitored their mRNA levels (**Figure 3C**). AZD4547 dose dependently impedes expression of E2F1 and CCND1. Together, the observations show that AZD4547 not only suppresses phosphorylation of TRK but also the downstream pathway of TRK in KM12(Luc).

### Anti-Tumor Activity of AZD4547 Against KM12(Luc) (TPM3-NTRK1)

**Figure 4:**
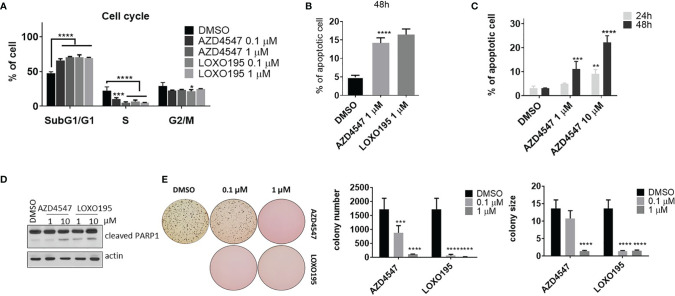
Anti-tumor effect of AZD4547 on KM12(Luc) (TPM3-NTRK1). **(A)** AZD4547 induces SubG1/G1 arrest. KM12(Luc) cells were treated with the indicated substances for 24 h. After fixation with 70% ethanol, cells were stained with PI containing RNase. Then, DNA contents of cells were analyzed by using flow cytometry. (average ± SD, n=3, two-way Anova; Dunnett’s multiple comparisons test; * < 0.05 *** < 0.001, ****p < 0.0001) **(B)** Apoptosis is induced by AZD4547 and LOXO195. (average ± SD, n=3 one-way Anova; ****p < 0.0001) **(C)** Time and dose dependent increases of apoptotic cells caused by AZD4547 treatment. (average ± SD, n=3 one-way Anova; ** <0.01, *** < 0.001 ****p < 0.0001) **(D)** Apoptotic marker, cleaved PAR-1 is up-regulated by AZD4547. **(E)** AZD4547 suppresses anchorage independent growth of KM12(Luc). (average ± SD, n=5 one-way Anova; *** < 0.001, ****p < 0.0001).

Several *in vitro* experiments were conducted to evaluate the anti-tumor activity of AZD4547 in KM12(Luc) (TPM3-NTRK1). First, we examined the effect of AZD4547 on the cell cycle of KM12(Luc) (**Figure 4A** and **Supplementary Figure 3A**). Significant SubG0/G1 arrest (P < 0.001) was observed to occur in association with AZD4547 or LOXO195 treatment for 24 h. This result is consistent with the finding (**Figure 3C**) that AZD4547 suppresses CCND1 expression, which is associated with the G1-S transition in the cell cycle (45). In addition, the number of apoptotic cells also increases after treatment with 1 µM of AZD4547 for 48 h, which is comparable to the increase brought about by LOXO195 (**Figure 4B** and **Supplementary Figure 3B**). Furthermore, AZD4547 induces apoptosis of KM12(Luc) in a time and dose dependent manner (**Figure 4C** and **Supplementary Figure 3C**). Upregulation of cleaved PARP1 by AZD4547 is also observed in KM12(Luc) (**Figure 4D**). Next, we determined the ability of AZD4547 to suppress tumorigenesis of KM12(Luc) by assessing its ability to block anchorage independent growth. The results of a soft agar assay in KM12(Luc) cells (**Figure 4E**) showed that complete suppression of colony formation and a reduction in size occurs after 14 d treatment with AZD4547. In order to show that the anti-tumor effect of AZD4547 against KM12(Luc) is caused by inhibition of TRKs rather than FGFR, we monitored mRNA levels of FGFR1/2 and NTRK1/2 in KM12(Luc) (**Supplementary Figure 1B**). The finding that FGFR1 is hardly expressed and NTRK1 mRNA expression is high provides clear evidence for the anti-tumor effect of AZD4547 in KM12(Luc) (TPM3-NTRK1).

### *In Vivo* Efficacy of AZD4547 in the KM12(Luc) Xenograft Model

**Figure 5:**
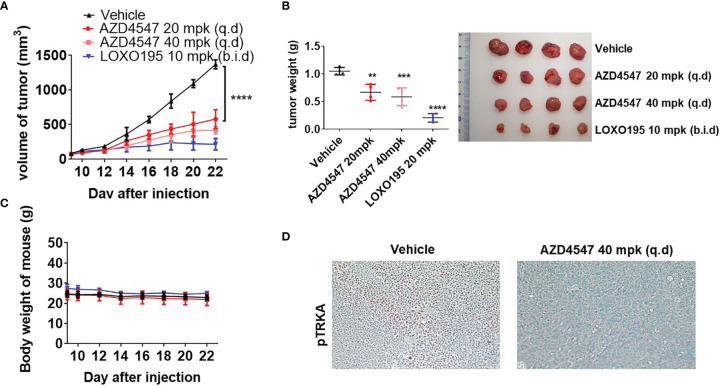
*In vivo* efficacy of AZD4547 in the KM12(Luc) xenograft model. **(A)** AZD4547 treatment significantly delayed tumor growth. KM12(Luc) implanted mice were administered by oral gavage (p.o, q.d for vehicle and AZD4547, b.i.d for LOXO195) with the indicated dosage (n = 4, Allow indicates the administration starting day. Tukey’s multiple comparisons test, **** < 0.0001). Tumor volume (mm^3^) was measured once every 2 d. Data was plotted as mean ± SD (n=4) **(B)** Tumor weight was measured at the end of the experiment. (Dunnett’s multiple comparisons test, ** < 0.005, *** < 0.001, **** < 0.0001) **(C)** Body weight was monitored during the experiment. **(D)** Immunohistochemical staining of paraffin-embedded tumor sections using phospho-TRKA antibody. IHC images were obtained at 200X magnification. The data represent tumor tissue from each group.

The results of *in vitro* studies that show that AZD4547 has comparable activities to those of LOXO195 led us to examine the *in vivo* efficacy of AZD4547 in the KM12(Luc) xenograft model. The balb/c nude mice bearing KM12(Luc) xenograft tumor were randomly grouped in 4 cohorts (vehicle, AZD4547 20 mpk, AZD4547 40 mpk or LOXO195 20 mpk, n = 4) and each group was orally administrated (q.d for vehicle or AZD4547, b.i.d for LOXO195). The results show that tumor growth in the AZD4547 treated group is remarkably delayed compared to that of the vehicle treated group (**Figures 5A, B**). During the time interval in which the tumor size of the vehicle treated group reached 1500 mm^3^, the tumor size of the group administered with 20 mpk of AZD4547 reached 600 mm^3^ and that of the 40 mpk AZD4547 treated group reached <500 mm^3^. In addition, while the average tumor weight of vehicle treated group was around 1.2 g, that of the 20 mpk AZD4547 treated group was 0.78 g and that of the 40 mpk AZD4547 treated group was 0.69 g. Reduction in body weight did not occur in all groups (**Figure 5C**). To confirm that the reduced tumor sizes are caused by TRK inhibition, we performed immunohistochemical staining of tissues of tumors from the vehicle and 40 mpk AZD4547 treated group (**Figure 5D**). In contrast to that in the vehicle group, decreased phosphorylation of TRKA took place in the 40 mpk AZD4547 treated group, which indicates that AZD4547 effectively inhibits phosphorylation of TRKA *in vivo*.

## Discussion

Drug repurposing is an effective approach to discover a new indication for an existing drug. Compared with the traditional drug development process, comprised of five stages including discovery, preclinical evaluation, safety review, clinical studies, and FDA review and post-market safety monitoring, the drug repurposing approach requires only four stages involving compound identification, acquisition and development, and FDA post-market safety monitoring. Thus, the drug repurposing protocol leads to time and money savings (4). A number of existing drugs developed employing this strategy are under clinical trials or have been remarketed for different indications (46).

As background for the drug repurposing study described here, we made the novel observation that AZD4547 is active against FGFR2-mutated endometrial cancers (43). AZD4547 is a selective kinase inhibitor targeting FGFR1/2/3 (28). The high *in vitro* potency of AZD4547 against cancer cells expressing FGFR mutations, including non-small cell lung cancer, breast cancer and gastric cancer cell lines, stimulated several clinical studies on patients who have a FGFR mutation. However, this substance has not yet received FDA approval (34). It is of note that studies of AZD4547 carried out to date focused only on tumor cell lines derived from FGFR mutants. This realization suggests the need to initiate a drug repurposing effort to probe the potential effects of AZD4547 on sensitive cancer cell lines other than just FGFR-mutant related cancers.

To investigate the cancer cell growth inhibitory activity of AZD4547, we examined its anti-proliferative activity against 64 cancer cell lines. To our surprise, the results of this exploratory investigation revealed that AZD4547 strongly inhibits proliferation of KM12(Luc) harboring the TPM3-TRKA rearrangement, which is known to be tumor sensitive for TRKA kinase inhibition (35). Considering the fact that the FGFR inhibitor BGJ-398 has no effect on proliferation of KM12(Luc), we assumed that AZD4547 directly inhibits TRKA kinase activity. Using an *in vitro* kinase assay, we unequivocally demonstrated for the first time that AZD4547 exhibits remarkable *in vitro* potencies against TRKA/B/C (IC_50_ = 8.8 nM for TRKA, 7.6 nM for TRKB, and 1.0 nM for TRKC). In regard to interactions that govern the affinity of AZD4547 for the TRKAs, it is known that three hydrogen bonds between the pyrazole amide of AZD4547 and the hinge region (E562 and A564) of FGFR1 are critical for binding (47). TRKA/B/C also have an EYM motif which corresponds to E562-Y-A564 of FGFR1/2/3 (3), suggesting that AZD4547 bind to the EYM motif of TRKA/B/C.

NTRK genes encoding the neurotrophin receptors TRKA, TRKB and TRKC are oncogenic drivers of various tumor types. Intra/inter-chromosomal rearrangements of the NTRK gene with partner genes including TPM3 and ETV6 lead to constitutive activation of TRK kinases. Even though the NTRK fusion gene (<1%) is infrequently present in solid tumors, first generation TRK inhibitors like LOXO101 display remarkable response rates (>75%) in patients in which this fusion is found (8). The high levels of response led to FDA approval of LOXO101 for the treatment of NTRK fusion positive tumors, regardless of tumor histology.

Acquired resistance to first generation TRK inhibitors has been observed and attributed to amino acid substitution at the solvent front region, activation loop or gate keeper residue of TRKA. Next generation TRK inhibitors such as LOXO195 and TRX-005, which inhibit many of the aforementioned TRK mutants, are currently being evaluated in clinical studies for safety and efficacy (8). To determine if AZD4547 is capable of inhibiting those mutants, we utilized ETV6-TRKA/B transformed Ba/F3 cell lines. Unfortunately, the results show that AZD4547 has little effect on growth of Ba/F3 cells containing the clinically relevant TRKA mutants including G595R, G667A, G667S and G667C. Type II-NTRK inhibitors (e.g. altiratinib, caboztinib, and foretinib) are more potent than type I inhibitors against entractibnin and larotrectinib-resistant TRKA mutations (G667C, V573M and F589L) (48). Thus, it might be possible to increase potency against drug resistant mutants by modifying AZD4547 (a type I inhibitor) to make it structurally more like type II kinase inhibitors.

The anti-tumor activity of AZD4547 was also evaluated in KM12(Luc) colon cancer cells harboring the TPM3-NTRK1 fusion gene. AZD4547 dose dependently blocks phosphorylation of TRKA/B and its downstream molecules including PLC-gamma and AKT. Like that with other TRK inhibitors, treatment with AZD4547 for 24 h results in down regulation of DUSP6, ETV1, E2F and CCND1. Because CCND1 is related with G1-S transition in the cell cycle, we performed cell cycle analysis after 24 h AZD4547 treatment. The results show that AZD4547 leads to Sub G1/G1 arrest of KM12(Luc) cells in a dose dependent manner. In addition, AZD4547 induces apoptosis and blocks tumorigenesis in the soft agar assay. A comparison of mRNA levels by using RT-PCR shows that expression of FGFR1/2 in KM12(Luc) cells is very low. Phosphorylation of FGFR1/2 in KM12(Luc) is undetectable by using western blot (data not shown), indicating that the anti-tumor effect of AZD4547 is a consequence of inhibition of TRKA/B rather than FGFR. *An in vivo* efficacy test in the KM12(Luc) xenograft model demonstrates that AZD4547 blocks tumor growth by inhibiting phosphorylation of TRKA. The anti-tumor activities of AZD4547 in this study are overly lower than those of LOXO195, which is a frontrunner inhibitor against TRKs.

## Conclusion

In summary, the above effort focused on finding therapeutic applications of AZD4547 other than those associated with FGFR inhibition. A screen of 64 cancer cell lines showed that AZD4547 significantly suppresses proliferation of KM12(Luc) cells *via* inhibition of TRKA kinase activity. Moreover, we found that AZD4547 has a potency (GI_50_ = 49.74 nM) that is comparable to that of LOXO101 (GI_50_ = 31.64 nM), an approved NTRK inhibitor. It is worth noting that LOXO101 is the first approved drug to treat any cancers harboring certain mutations. We also demonstrated that AZD4547 significantly blocks proliferation of Ba/F3 cells transformed with TRKA (66 nM of GI_50_) and TRKB (100 nM of GI_50_). However, AZD4547 has no anti-proliferative activity against Ba/F3 cells harboring clinically relevant TRK mutants. In addition, AZD4547 not only suppresses phosphorylation of TRK, it also perturbs the downstream pathway of TRK in KM12(Luc) cells. Furthermore, we demonstrated that AZD4547 exhibits an anti-tumor effect on KM12(Luc) by perturbing the TRKA pathway including phosphorylation of PLC-gamma and expression of DUSP6 and ETV1. Apoptotic cells are also increased by AZD4547 treatment which in a manner that is comparable to that of LOXO195, a promising next-generation NTRK inhibitor. AZD4547 induces complete suppression of colony formation and reduces tumor size in a soft agar assay. Furthermore, AZD4547 induces apoptosis of KM12(Luc) in a time and dose dependent manner (**Figure 4C** and **Supplementary Figure 3C**). Finally, AZD4547 potently delays tumor growth in a KM12(Luc) xenograft model. The combined results of the investigation lead to the unprecedented suggestion that AZD4547 exhibits therapeutic activity against NTRK mutant derived cancer cells by inhibiting TRKA signaling.

## Data Availability Statement

The original contributions presented in the study are included in the article/**Supplementary Material**. Further inquiries can be directed to the corresponding author.

## Ethics Statement

The animal study was reviewed and approved by Korea Institute of Science and Technology.

## Author Contributions

HC designed and performed experiments and wrote the manuscript. NK performed experiments. TM provided KM12(Luc) cell lines. TS conceived, proofread, and edited the manuscript. All authors contributed to the article and approved the submitted version.

## Funding

This study was supported by NRF-2021R1A2C3011992 from the National Research Foundation in Korea, Korea Institute of Science and Technology (KIST), Brain Korea 21 Project.

## Conflict of Interest

The authors declare that the research was conducted in the absence of any commercial or financial relationships that could be construed as a potential conflict of interest.

## Publisher’s Note

All claims expressed in this article are solely those of the authors and do not necessarily represent those of their affiliated organizations, or those of the publisher, the editors and the reviewers. Any product that may be evaluated in this article, or claim that may be made by its manufacturer, is not guaranteed or endorsed by the publisher.
