# Supplementary material for: Anti-Tumor Activity of AZD4547 Against NTRK1 Fusion Positive Cancer Cells Through Inhibition of NTRKs
Source: Front Oncol. 2021 Nov 1;11:757598. doi: 10.3389/fonc.2021.757598 (PMC8591201; doi:10.3389/fonc.2021.757598)
Supplement: Supplementary file 1 [file DataSheet_1.docx]

Supplementary Figures

**Supplementary Figure 1:** (A) inhibitory activity of FGFR inhibitors in KM12 cells. KM12 cells were treated with the indicated inhibitors at 10 μM for 2 h. Phosphorylation of TRKA/B were determined by western blot analysis. (B) mRNA level of FGFR 1/2 and NTRK1/2 respectively in KM12 and AN3CA were compared by RT-PCR. After total RNA of KM12 and AN3CA were isolated, cDNA was synthesized. Indicated gene were amplified.

**Supplementary Figure 2:** Established Ba/F3 cells expressing TrkA, TrkA point mutation and TrkB were validated by RT-PCR and western blot. (A) Cartoon shows the DNA construct for establishing Ba/F3 cells in this study. The kinase domain of NTRK1 (434-790) or NTRK2 (455-822) respectively was fused with ETV6 (1-336) for the constitutive activation and followed mutagenesis PCR for making indicated point mutation. (B-C) cDNA isolated from indicated Ba/F3 cells were synthesized and amplified followed sequencing analysis. (D) The phosphorylation TrkA/B level of indicated Ba/F3 cell were determined by western blot.

**Supplementary Figure 3.** Anti-tumor activity of AZD4547 in KM12 (TPM3-NTRK1). (A) The representative FACS image for the effect of AZD4547 on the cell cycle. After 24 h incubation with indicated compounds, cell stained with PI. The DNA contents of each cell were determined by using FACS. (B-C) The representative FACS image for the effect of AZD4547 on apoptosis induction. KM12 cells were incubated with indicated compounds for 24 h or 48 h and subjected for the FACS analysis.

**Supplementary Figure 4.** Paraffin-embedded tumor sections isolated from Vehicle or AZD4547 administered mice were stained by phospho-TRKA antibody. IHC images were obtained at 40X magnification.

**Supplementary Tables**

**Table S1.** GI_50_s of AZD4547 against 64 cancer cell lines.

| cell lines | GI_50_ (µM) *^a^* | cell lines | GI_50_ (µM) *^a^* | cell lines | GI_50_ (µM) *^a^* |
| --- | --- | --- | --- | --- | --- |
| KM12-Luc | 0.11 | U138 | 8.48 | 8505C | 14.66 |
| AN3-CA | 0.20 | PANC-1 | 8.65 | HCT15 | 15.78 |
| RT-112 | 0.42 | U-87-MG | 8.94 | SK-MEL-30 | 16.54 |
| U-2-OS | 0.43 | HL-60 | 9.31 | HCC70 | 16.58 |
| MV-4-11 | 0.93 | J82 | 9.54 | K-562 | 17.05 |
| GIST-T1 | 1.09 | HT-29 | 9.63 | NCI-H2228 | 17.25 |
| Molm-14 | 1.25 | PC-3 | 10.55 | CAPAN-1 | 17.48 |
| HuH-7 | 1.35 | DU-145 | 10.70 | HEP3B | 19.70 |
| A172 | 2.18 | NCI-H23 | 10.82 | MDA-MB-231 | 20.90 |
| RS4-11 | 3.24 | NCI-H1666 | 10.85 | LNCaP | 22.70 |
| HeLa | 4.38 | MKN28 | 10.91 | SNU484 | 24.55 |
| OVCAR-8 | 4.97 | SW620 | 11.04 | NCI-H2087 | 27.35 |
| T98G | 5.21 | C8161 | 11.12 | DLD-1 | 27.47 |
| MDA-MB-453 | 5.36 | A375 | 11.40 | NCI-H1975 | 28.92 |
| NCI-H417 | 5.99 | MDA-MB-468 | 11.50 | HEC1A | 35.60 |
| KG-1 | 6.27 | U251 | 11.55 | NCI-H1437 | 40.33 |
| HTH-7 | 6.44 | A549 | 11.61 | UMUC3 | 46.30 |
| MOLT-4 | 6.59 | MKN45 | 11.99 | NCI-H2009 | 50.00 |
| OCI-AML3 | 6.75 | MIA-PaCa-2 | 12.18 | AGS | 58.09 |
| HCT-116 | 7.13 | U937 | 12.21 | NCI-H1299 | 60.57 |
| HCC-78 | 7.17 | SK-HEP-1 | 12.58 |  |  |
| MCF7 | 7.84 | SW480 | 14.07 |  |  |

*^a^* GI_50_ is the concentration at which half-maximal growth inhibition occurs. The cells were treated with AZD4547 for 72 h in a dose escalation manner. A CellTiter-Glo assay was performed to assess cell viability. All experiment was performed in duplicate.

**Table S2.** GI_50_s of AZD4547 against Ba/F3 harboring NTRK mutations.

|  | LOXO105 *^a^* | LOXO195 *^a^* | AZD4547 *^a^* |
| --- | --- | --- | --- |
| TRKA | 9.7 ± 5.1 | 1.7 ± 0.8 | 66.0 ± 11.5 |
| TRKA (G595R) | > 2000 | 23.0 ± 9.5 | > 2000 |
| TRKA (G667S) | 867.6 ± 603.0 | 35.5 ± 2.5 | > 2000 |
| TRKA (G667C) | > 2000 | 319.2 ± 22.8 | > 2000 |
| TRKA (G667A) | 264.8 ± 57.6 | 24.1 ± 8.3 | 1223.5 ± 603.2 |
| TRKB | 11.7 ± 0.5 | 1.4 ± 0.8 | 104.3 ± 6.1 |
| Parental | > 2000 | > 2000 | > 2000 |

*^a^* GI_50_ is the concentration at which half-maximal growth inhibition occurs. The cells were treated with AZD4547 for 72 h in a dose escalation manner. A CellTiter-Glo assay was performed to assess cell viability. All experiment was performed in duplicate.
