# Supplementary figures and images for: Anti-Tumor Activity of AZD4547 Against NTRK1 Fusion Positive Cancer Cells Through Inhibition of NTRKs
Source: Front Oncol. 2021 Nov 1;11:757598. doi: 10.3389/fonc.2021.757598 (PMC8591201; doi:10.3389/fonc.2021.757598)

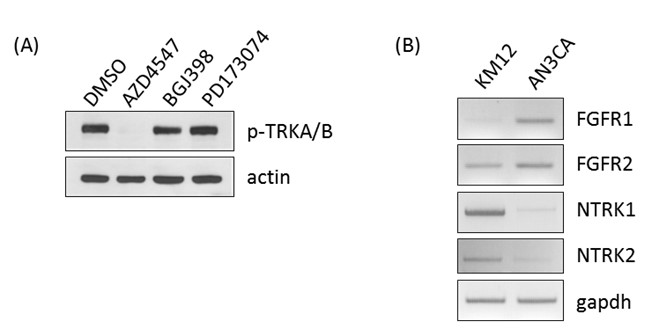

Supplement: Supplementary file 2 [file Image_1.jpeg]

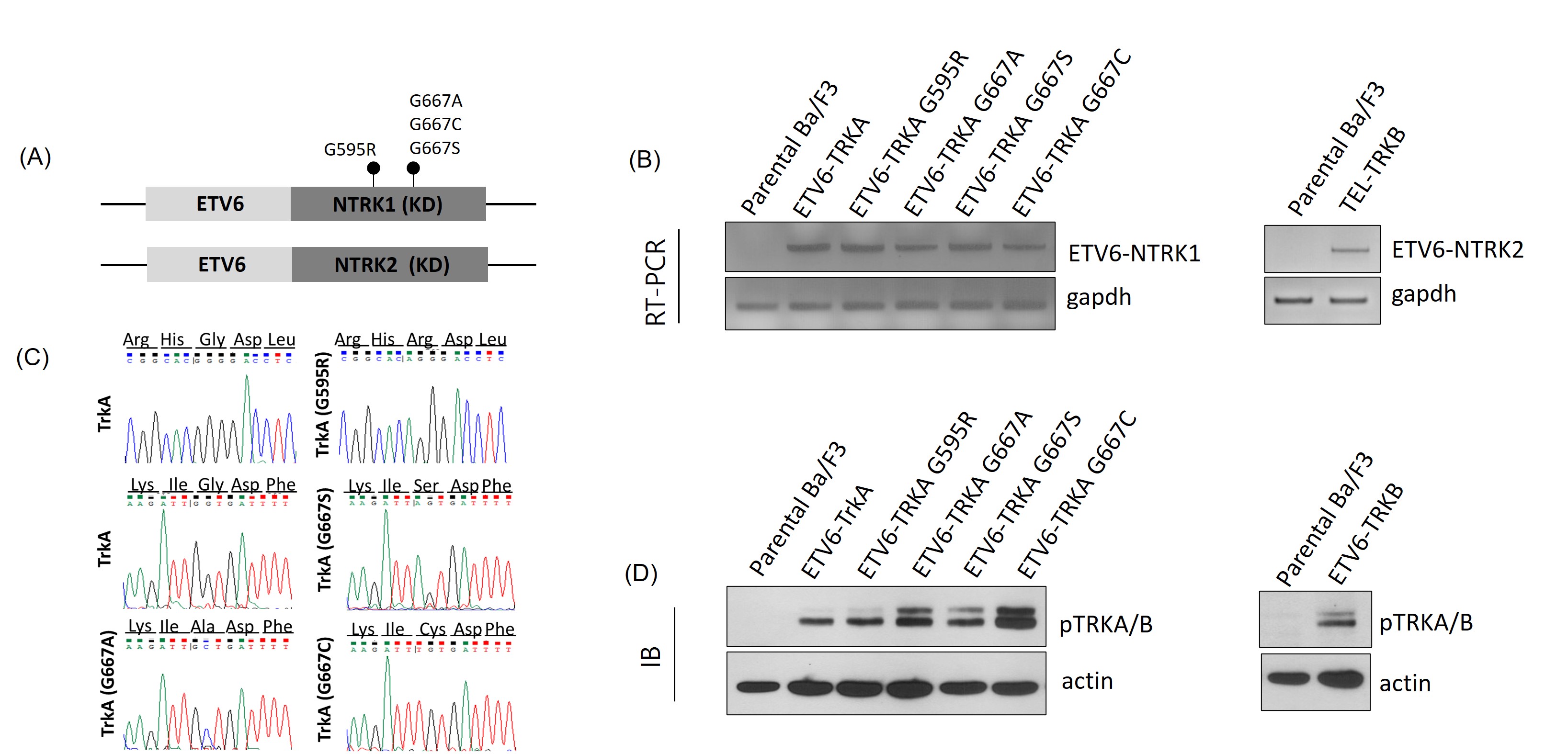

Supplement: Supplementary file 3 [file Image_2.jpeg]

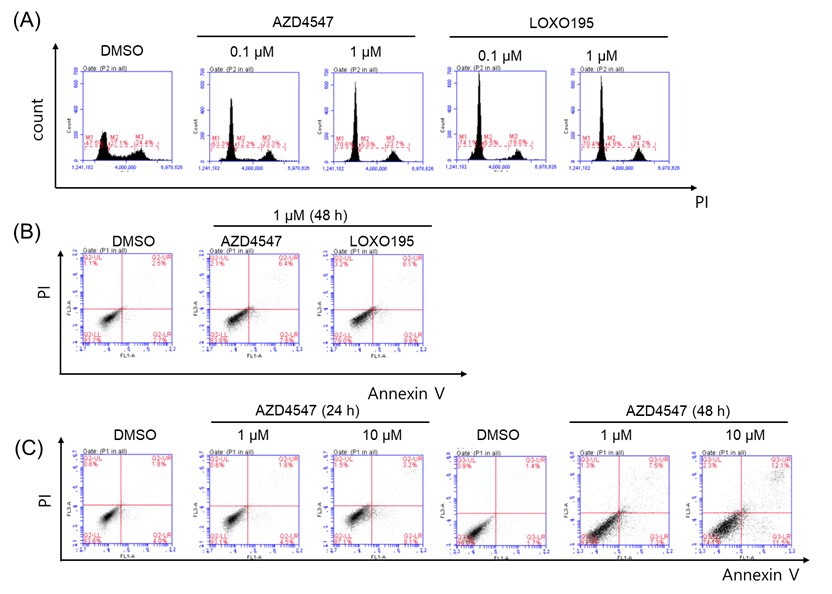

Supplement: Supplementary file 4 [file Image_3.jpeg]

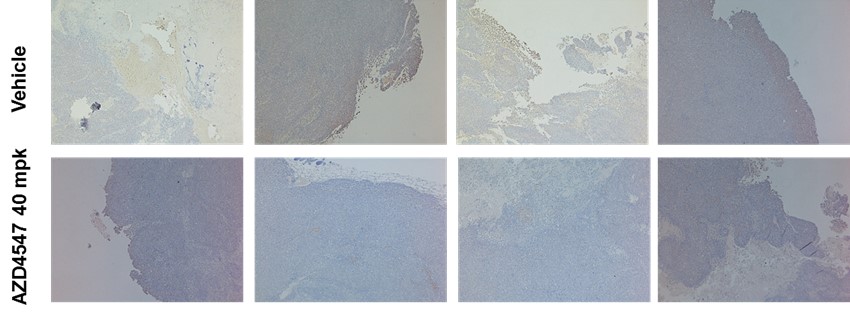

Supplement: Supplementary file 5 [file Image_4.jpeg]
